# MARV: a tool for genome-wide multi-phenotype analysis of rare variants

**DOI:** 10.1186/s12859-017-1530-2

**Published:** 2017-02-16

**Authors:** Marika Kaakinen, Reedik Mägi, Krista Fischer, Jani Heikkinen, Marjo-Riitta Järvelin, Andrew P. Morris, Inga Prokopenko

**Affiliations:** 1 Department of Genomics of Common Disease, Imperial College London, London, W12 0NN UK; 2 Estonian Genome Center, University of Tartu, Tartu, 51010 Estonia; 3 Neuroepidemiology and Ageing (NEA) Research Unit, Imperial College London, London, W6 8RP UK; 4 Department of Epidemiology and Biostatistics, MRC-PHE Centre for Environment and Health, School of Public Health, Imperial College London, London, W2 1PG UK; 5 Center for Life Course Health Research, University of Oulu, 90014 Oulu, Finland; 6 Unit of Primary Care, Oulu University Hospital, 90220 Oulu, Finland; 7 Biocenter Oulu, University of Oulu, 90014 Oulu, Finland; 8 Department of Biostatistics, University of Liverpool, Liverpool, L69 3BX UK

**Keywords:** Rare variant analysis, Multi-phenotype analysis, High-dimensional data

## Abstract

**Background:**

Genome-wide association studies have enabled identification of thousands of loci for hundreds of traits. Yet, for most human traits a substantial part of the estimated heritability is unexplained. This and recent advances in technology to produce high-dimensional data cost-effectively have led to method development beyond standard common variant analysis, including single-phenotype rare variant and multi-phenotype common variant analysis, with the latter increasing power for locus discovery and providing suggestions of pleiotropic effects. However, there are currently no optimal methods and tools for the combined analysis of rare variants and multiple phenotypes.

**Results:**

We propose a user-friendly software tool MARV for Multi-phenotype Analysis of Rare Variants. The tool is based on a method that collapses rare variants within a genomic region and models the proportion of minor alleles in the rare variants on a linear combination of multiple phenotypes. MARV provides analyses of all phenotype combinations within one run and calculates the Bayesian Information Criterion to facilitate model selection. The running time increases with the size of the genetic data while the number of phenotypes to analyse has little effect both on running time and required memory. We illustrate the use of MARV with analysis of triglycerides (TG), fasting insulin (FI) and waist-to-hip ratio (WHR) in 4,721 individuals from the Northern Finland Birth Cohort 1966. The analysis suggests novel multi-phenotype effects for these metabolic traits at *APOA5* and *ZNF259*, and at *ZNF259* provides stronger support for association (*P*_TG+FI_ = 1.8 × 10^−9^) than observed in single phenotype rare variant analyses (*P*_TG_ = 6.5 × 10^−8^ and *P*_FI_ = 0.27).

**Conclusions:**

MARV is a computationally efficient, flexible and user-friendly software tool allowing rapid identification of rare variant effects on multiple phenotypes, thus paving the way for novel discoveries and insights into biology of complex traits.

**Electronic supplementary material:**

The online version of this article (doi:10.1186/s12859-017-1530-2) contains supplementary material, which is available to authorized users.

## Background

In the past decade, genetic locus discovery for human traits and diseases has been advanced via genome-wide association studies (GWAS). Recent improvements in technology to produce genotype data in a very cost- and time-effective manner and powerful easy-to-use software tools have played a major role in these advances, facilitating fast analysis of constantly increasing amounts of data. Clearly, the next advances in the field of genomics will be based on large-scale sequencing and other high-dimensional omics data. A key challenge for successful utilisation of these data lies, once again, in the availability of powerful methods and user-friendly software tools, thus enabling researchers to make rapid discoveries [1].

Large-scale sequencing efforts, such as the 1000 Genomes Project [2] or more recently the UK10K Project [3] and the Haplotype Reference Consortium [4], have enabled better characterization of variation in the human genome, especially in the low-frequency and rare variant range. Here, we denote all variants with minor allele frequency, MAF < 5%, by RVs. Imputation based on variant density detected by these projects yields high-quality genotype data even down to 0.01% allele frequency [5]. Large scale sequencing data generation encourages method and software development for elucidating RV effects, since traditional single-variant methods are underpowered to detect RV associations. Several methods and related software tools have been proposed, including burden tests using collapsing techniques, variance-component tests and combinations of the two [6].

There has also been increasing interest in addressing analysis of high-dimensional phenotypic and omic data, such as metabolomics, in relation to human genome variation. Multi-phenotype analysis (MPA), i.e. joint analysis of multiple phenotypes, is an example of recent developments in the field. Several methods and related software for single-variant MPA, including Bayesian and frequentist approaches, have recently been published [7]. The MPA approach is motivated by several factors: 1) it boosts power for locus discovery [8–11]; 2) it provides more precise parameter estimates [12]; and 3) it has biological advantages including the possibility to identify multi-phenotype effects, including pleiotropy [13], when one locus affects multiple phenotypes. The power improvement by the MPA approach is especially relevant from a computational point of view, because to enable the discovery of further loci for complex traits, the analyses will need to be based on hundreds of thousands of individuals, such as those available from the UK Biobank and other new large-scale efforts based on sequencing. Storage and computational load for such amounts of data will pose a challenge, and alternative strategies for boosting power for locus discovery other than that of increasing sample size, clearly bring an enormous advantage.

We propose a novel tool MARV for RV MPA, which enables joint analysis of both large-scale high-dimensional genomic and phenotypic data. It extends the burden test for RVs to high-dimensional phenotypic data by applying the MPA approach. Recently, methods designed for MPA of RVs have been proposed [14–16], but these have several limitations regarding scalability and ability to combine continuous and discrete phenotypes, and more importantly, the associated software: they either lack an easy user-interface or are computationally inefficient – key features to facilitate fast discoveries. Our software tool MARV enables analysis of both continuous and binary phenotypes, as well as genotyped, imputed or sequenced data. MARV is computationally efficient for large-scale data. From a user point of view, it enables standard formats of data as used in other GWAS software, and the analyses are run using a command line interface, also familiar from widely used GWAS software such as Plink [17] and SNPTEST [18], thus enabling researchers quickly and effortlessly to transit from the standard single variant, single phenotype GWAS to region-based analysis of multiple phenotypes.

## Implementation

The method on which MARV is based is briefly introduced in Methods, and is extensively described, including power simulations, elsewhere [19] (Methods). MARV is written in C++ and has a command line user-interface. A single run of MARV consists of just one step and the required input files, commands and the resulting output files are described below.

### Data input and commands

**Fig. 1:**
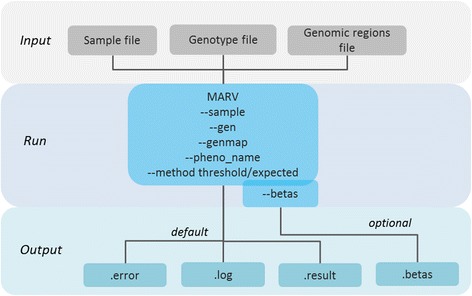
Workflow of a MARV run including required files, commands and resulting output files

**Fig. 2:**
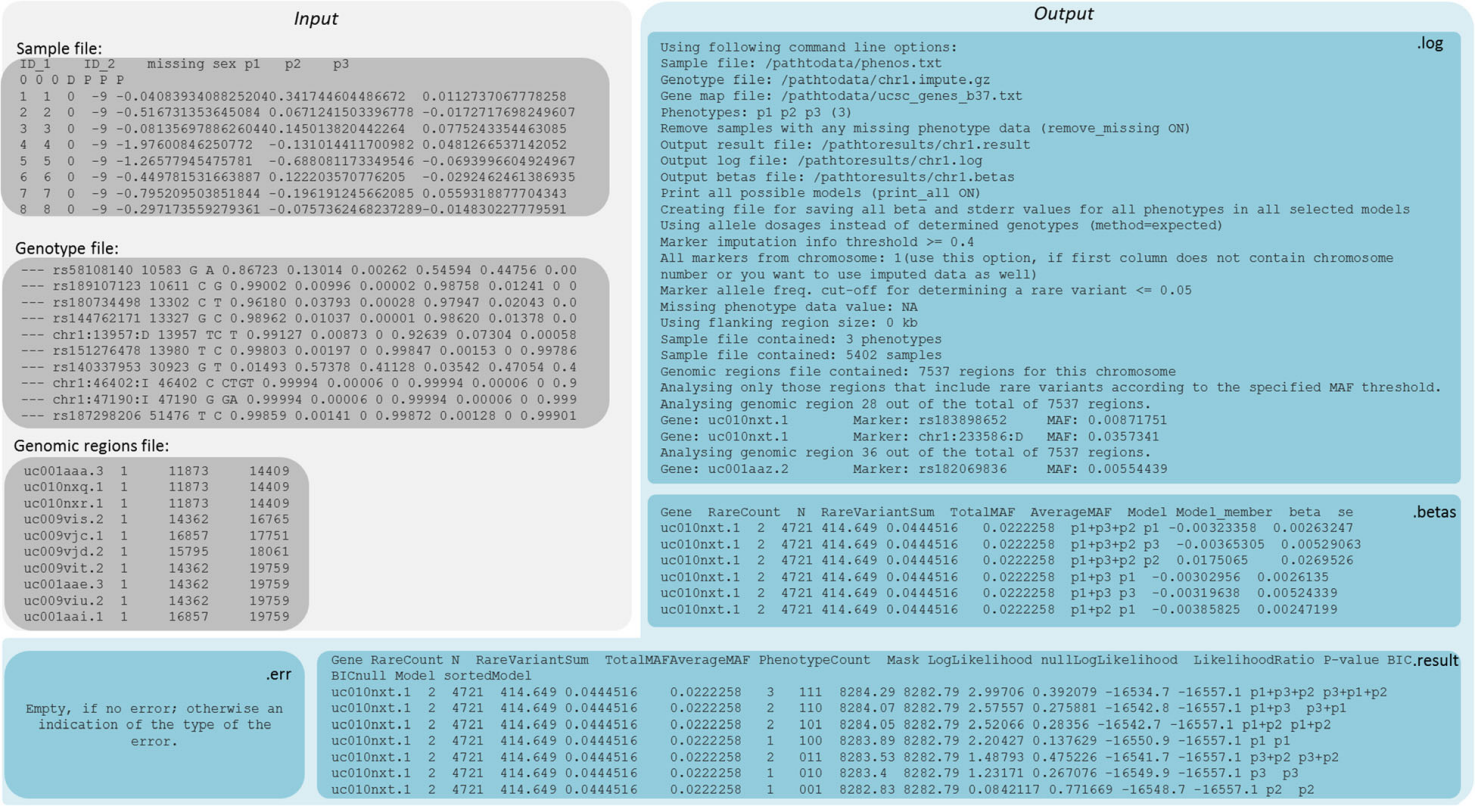
Examples of the required input file formats for MARV and the resulting output files

MARV requires three files for a successful run: sample, genotype and genomic region input files (Fig. 1). The sample and the genotype input files should be in the SNPTEST v2 [18] format. The genomic region file should contain three columns: the name of the region, and the start and the end positions for the region. It is important that the positions in this file correspond to the positions of the genotype file, i.e. the same genome build for these two should be used (Fig. 2).

The user then needs to specify the phenotypes to be analysed (--pheno_name), corresponsing to a column name in the sample file, and the method to use for the analysis, i.e. whether to analyse the genotype dosages derived by the software from the imputation probabilities (--method expected) or whether to use the thresholded genotypes based on a pre-defined cut-off (--method threshold, with a cut-off default 0.9 which can be changed with the --call_thres option) (Fig. 1). Additionally, the user may specify several other options, such as individuals or SNPs to extract or exclude from the analysis. It is important to specify the threshold used for the minor allele frequency (--rare_thresh, by default 0.05, which means that variants with minor alleles of frequency < 5% only will be included in the analysis). All the available options of the latest version of MARV can be found from the online manual of MARV.

### Data analysis

MARV works across the genome by going through the specified gene regions one by one. Based on the gene boundaries and desired rare variant cut-off, it calculates, for each individual, the proportion of minor alleles at rare variants within the region [20]. After this calculation is performed for all individuals, a linear regression is fitted using the proportion as the outcome and the listed phenotypes as its predictors. The likelihood contribution of each individual is further weighted by the number of successfully genotyped/imputed RVs in the region of interest. For each genomic region, weighted linear regression is performed for all different phenotype combinations, i.e. if a user specifies phenotypes *pheno_a* and *pheno_b*, three different models for the proportion are fitted with the following predictor combinations: 1) *pheno_a + pheno_b*, 2) *pheno_a*, 3) *pheno_b*. MARV calculates the Bayesian Information Criterion (BIC) for each model to help the user in identifying the best fitting phenotype combination.

### Output files

MARV produces three files by default:.error file,.log file and.result file (Fig. 1). The error file will be empty if the run was completed successfully; otherwise details about problems during the run are reported (Fig. 2). The log file will give specific details of the analysis, including the number of samples in the sample file and genotype file, and the number of phenotypes used for the analysis. It will also include the variants included for the analysis of each genomic region, along with their MAFs. The results file will include one row for results per each genomic region. If the user specifies printing of all the possible model combinations (--print_all) there will be as many rows per gene as there were different model combinations fitted. This file will inform the log likelihood and BIC of the model as well as the *P*-value for each model. We note that the *P*-value is uncorrected for any multiple testing. If the user is interested in the effect estimates and their standard errors for each of the model members, i.e. phenotypes included in the fitted model, a separate.betas file can be requested (--betas) (Figs. 1 and 2). A complete list of the columns in the output file with their meanings is provided in the online tutorial of MARV.

### Case study

To illustrate the use of MARV across the genome, we have applied it to data from the Northern Finland Birth Cohort 1966 (NFBC1966), which covers over 96% of all births in the two northernmost provinces of Finland in 1966 (*N* = 12,068 live-born children) [21]. We included data from 4,721 cohort members who had participated in the 31 year clinical examination and had genetic data as well as data on triglycerides (TG), fasting insulin (FI) and waist-to-hip ratio (WHR). The Ethics Committees of the University of Oulu and Northern Ostrobothnia Hospital District have approved the study. Individuals used for the analyses have provided written, informed consent.

Motivation for the selection of the traits comes from a common variant single-trait GWAS, which has shown an enrichment of FI associations among SNPs preselected on Metabochip for TG and waist phenotypes [22]. For the selected traits, we applied the following criteria: 1) FG: exclude non-fasting individuals and/or those having type 1 or 2 diabetes mellitus or on diabetes treatment or having fasting blood glucose ≥ 7 mmol/l and/or being pregnant, 2) TG: exclude non-fasting and/or individuals known to be on lipid lowering medication. We modelled each trait on sex, body mass index and the first three principal components derived from the genetic data to control for potential population structure. An inverse normal transformation was further applied to the residuals of WHR and TG to reduce skewness.

DNA was extracted from blood samples drawn after overnight fasting at the 31 year clinical examination. Genotyping was performed with the Illumina HumanCNV370DUO Analysis BeadChip platform at the Broad Institute, USA, with Beadstudio algorithm being used for genotype calling. Detailed genotyping and sample quality control (QC) of the first set of data have been reported before [23]. Additional samples were genotyped afterwards, resulting in 5,402 subjects and 324,896 SNPs available for analysis. The 1,000 Genomes Project “all ancestries” reference panel (March 2012) was used for imputation, resulting in ~38 M SNPs for analysis.

We analysed the transformed residuals in MARV with the method “threshold” (option -m threshold), i.e. genotypes with probability of 0.95 or higher were considered called, whilst all others were considered missing. The gene list from the University of California Santa Cruz (UCSC, NCBI genome sequence build 37, hg19) [24] was used to define gene regions, and a level of significance of 1.67 × 10^−6^ was adopted based on a Bonferroni correction for 30,000 genes. We analysed all variants irrespective of their annotation across autosomal chromosomes using the following cut-offs: MAF < 5% and imputation quality > 0.4.

## Results and discussion

### Case study

**Fig. 3:**
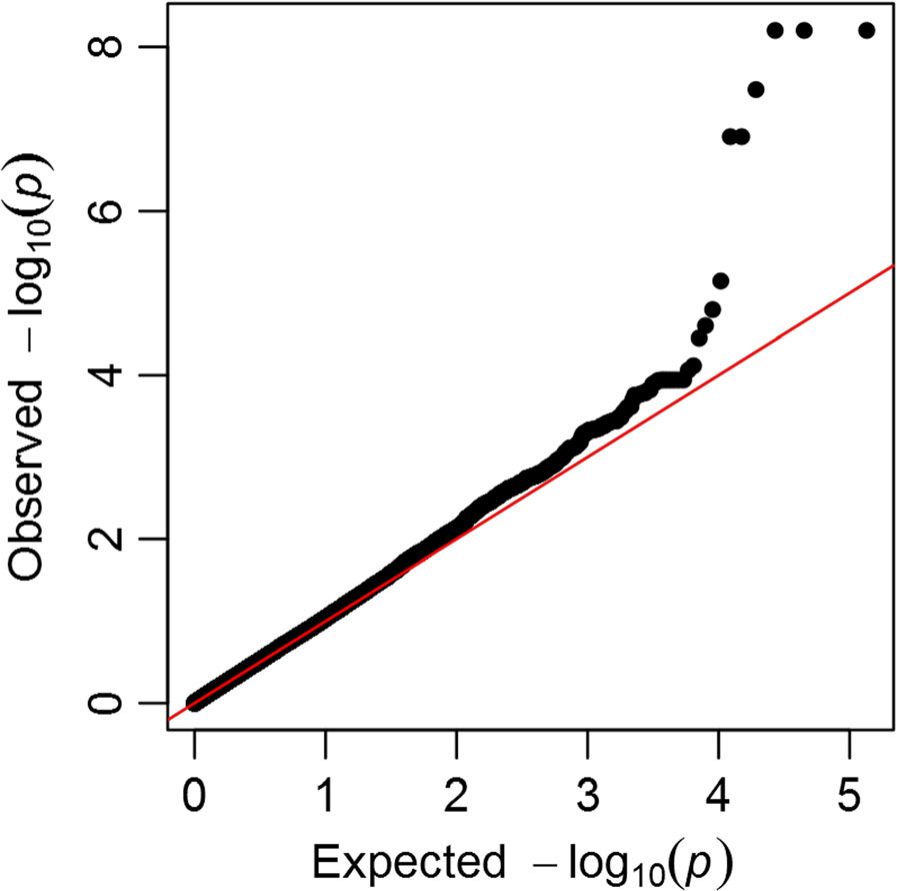
QQ-plot of MARV analysis results on triglycerides, fasting insulin and waist-to-hip ratio in the NFBC1966

**Fig. 4:**
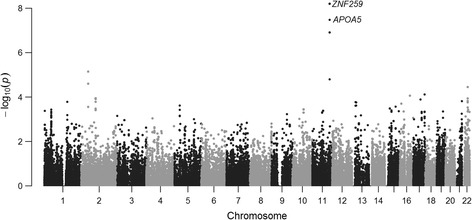
Manhattan plot of MARV analysis results on triglycerides, fasting insulin and waist-to-hip ratio in the NFBC1966. Genes reaching genome-wide significance (*P* < 1.67 × 10^−6^) are annotated

The three selected phenotypes, FI, TG and WHR, were modestly correlated with each other (R_FI_TG_ = 0.37, R_FI_WHR_ = 0.18, R_TG_WHR_ = 0.19). The multi-phenotype analysis of the three phenotypes revealed genome-wide significant associations covering two gene regions on chromosome 11: at *APOA5* (apolipoprotein A-V) and at *ZNF259* (the zinc finger protein 259, also known as *ZPR1*) genes (Table 1, Figs. 3 and 4). Besides the full model, MARV also provides parameter estimates and tests of associations for each phenotype combination, including the single phenotype models. Therefore, we were able to compare the results from the joint analysis against traditional single phenotype analyses. Additionally, the BIC provided by MARV for each sub-model served for selection of the phenotype combination providing the best fit. At *APOA5*, the best fitting model according to BIC contained TG only (*P* = 2.0 × 10^−7^), while at *ZNF259,* the model with FI and TG provided the lowest BIC and hence support for the best fit (*P* = 1.8 × 10^−9^) (Table 1). The model with FI and TG provided a lower *P*-value compared to those obtained from univariate models (*P*_TG_ = 6.5 × 10^−8^ and *P*_FI_ = 0.27), suggesting that at least the association with FI would have been missed in univariate analyses. The effects of TG and FI on the rare allele load were in opposite directions: while the increase in TG levels was associated with a greater proportion of minor alleles within *ZNF259*, the opposite was true for FI. This was true also for the univariate models (Table 1). All results files outputted by MARV are available as Additional files 1, 2, 3 and 4.

**Table 1:** Results for loci reaching genome-wide significance in the multi-phenotype rare variant analysis of NFBC1966 (*N* = 4,721). Regression coefficients with their standard errors (SE) are reported, followed by the *P*-value and the Bayesian Information Criterion (BIC) for the analysed model. TG, triglycerides; ln(FI), natural logarithm transformed fasting insulin; WHR, waist-to-hip ratio

	*APOA5* (Chr 11: 116,660,086-116,663,136)		*ZNF259* (Chr 11: 116,649,276-116,658,739)	
Model	β (SE)	*P*-value; BIC	β (SE)	*P*-value; BIC
TG + ln(FI) + WHR, full model ^a^		3.32 × 10^−8^; −19877.3		6.3 × 10^−8^; −25069.6
TG	0.011 (0.002)	-	0.007 (0.001)	-
ln(FI)	−0.010 (0.004)	-	−0.008 (0.002)	-
WHR	0.027 (0.019)	-	0.010 (0.011)	-
TG + ln(FI)		2.00 × 10^−8^; −19883.5		1.8 × 10^−9^; −25077.3
TG	0.011 (0.002)	-	0.007 (0.001)	-
ln(FI)	−0.010 (0.004)	-	−0.007 (0.002)	-
TG + WHR		3.34 × 10^−7^; −19877.9		4.1 × 10^−7^; −25066.5
TG	0.009 (0.002)	-	0.005 (0.001)	-
WHR	0.020 (0.019)	-	0.005 (0.001)	-
ln(FI) + WHR		0.08; −19853.1		0.10; −25427.6
ln(FI)	−0.003 (0.004)	-	−0.003 (0.002)	-
WHR	0.041 (0.019)	-	0.019 (0.011)	-
Univariate ^b^				
TG	0.009 (0.002)	9.15 × 10^−8^; −19885.1	0.005 (0.001)	6.5 × 10^−8^; −25074.7
ln(FI)	−0.002 (0.003)	0.62; −19856.8	−0.002 (0.002)	0.27; −25046.7
WHR	0.038 (0.019)	0.04; −19860.7	0.016 (0.011)	0.16; −25047.5

^a^ For a genome-wide joint analysis, the level of significance is *P* < 1.67 × 10^−6^ after Bonferroni correction for 30,000 genes

^b^ For univariate analysis, the level of significance is *P* < 5.56 × 10^−7^ after Bonferroni correction for 30,000 genes and three phenotypes

Common variants at these two identified genes have previously been associated with TG, total cholesterol, high-density lipoprotein, low-density lipoprotein, apolipoprotein A1 and B, coronary heart disease, coronary artery disease, plasma viscosity, Lp-PLA2 activity, prostate cancer, and circulating vitamin E levels [25–33]. A recent large-scale GWAS also reported RV associations at *ZNF259* with triglyceride levels [34]. Our analysis pointed to multi-phenotype effects with TG and FI. A recent study in Japanese individuals showed evidence for associations between variation in *ZNF259* and type 2 diabetes [35], making this locus of interest for further investigation in the pathogenesis of the disease. Interestingly though, in our MPA the effects of TG and FI on the rare allele load at *ZNF259* were in opposite directions, contrary to our expectations, since elevated TG levels usually correlate with elevated rather than decreased FI levels.

### Running time and memory

We measured running time and memory usage of MARV by performing additional analyses on the NFBC1966 data with different number of individuals, phenotypes and on different sized chromosomes. For these analyses, we used 2,405 and 4,809 (i.e. ~double the first) individuals with complete data on eight continuous phenotypes. We analysed a combination of two, four and eight continuous phenotypes and used 1000 Genomes imputed chromosomes 1 and 22 data for the association analyses. All analyses were run and their performance data were collected using Imperial College HPC Cluster. Compute nodes were equipped with Intel(R) Xeon(R) CPU E5-2620 v2 @ 2.10GHz machine.

The results are summarised in Table 2. We observe that the size of the genomic region to be analysed notably affects the running time. However, there is not a linear relationship between the number of phenotypes and the computation time required. For example, the increase in time for chromosome 1 is just under 3 h (17% from original time) even when the number of phenotypes is doubled from four to eight and the number of models to be fitted is more than 20-fold. Doubling the sample size roughly triples the runtime.

**Table 2:** Computational time and peak memory usage of MARV by varying sample size, chromosomal size and number of phenotypes

Number of phenotypes (number of fitted models)	Chr 1 (249 Mbp)	Chr 22 (35 Mbp)
*N* = 2,405	h:min:s (memory)	h:min:s (memory)
2 (3)	4:06:54 (215 MB)	00:38:04 (260 MB)
4 (15)	3:51:23 (215 MB)	00:38:37 (260 MB)
8 (63)	5:07:11 (215 MB)	00:55:58 (260 MB)
*N* = 4,809	h:min:s (memory)	h:min:s (memory)
2 (3)	14:47:34 (780 MB)	02:26:00 (500 MB)
4 (15)	13:40:11 (780 MB)	02:26:10 (600 MB)
8 (63)	17:26:08 (780 MB)	03:03:00 (600 MB)

The memory usage of MARV is more related to the size of the genetic data and number of individuals to analyse rather than the number of phenotypes to analyse. In our example, the peak memory usage was almost constant for all chromosome 1 and 22 analyses when the sample size remained the same, independent of the number of phenotypes in the model (Table 2). Considering the size differences of these two chromosomes (Table 2), we note that the increase in memory usage is not linear, however.

## Conclusions

Our novel tool MARV allows for RV analysis of multiple phenotypes in a computationally efficient and user-friendly manner. The data input formats and the command line interface familiar from widely-used GWAS software will offer researchers a quick setup for the analyses. Moreover, the feature of analysing all phenotype combinations within one run and the calculation of BIC to help in model selection will pave the way for rapid discoveries and novel insights into biology of complex traits.

## Methods

### Statistical model

MARV is based on a so-called “reverse regression” approach, i.e. as compared to the standard GWAS in which the phenotype is the outcome and the genotype the predictor, this scenario is reversed in MARV. By using the genetic data as the outcome, we enable assessment of associations with multiple phenotypes simultaneously through the use of simple linear regression. While the “reverse regression” approach has been proposed for single genetic variants with the risk allele count or allele dosage being the outcome [36, 37], MARV uses a mutational load (burden) of risk alleles at RVS as the outcome. That is, the outcome is the proportion of RVs at which minor alleles are carried by individuals within a genomic region. This proportion is then modelled as a linear combination of *K* phenotypes. Mathematically, if *r*_*i*_ is the number of minor alleles at RVs and *n*_*i*_ is the total number of RVs, the model becomes:

$$ {r}_i{n_i}^{\mathit{\hbox{-}} 1} = \upalpha +\boldsymbol{\upbeta} {\mathbf{y}}_{\mathrm{i}} + {\upvarepsilon}_{\mathrm{i}}, $$

where *r*_*i*_ *n*_*i*_^*−1*^ is the proportion of minor alleles for *i*th individual, **y**_*i*_ is a vector of phenotype data for individual *i*, with corresponding regression coefficients **β** = [β_1_,…, β_K_], and ɛ_i_ ~ MVN(0,ơ^2^), ơ^2^ being a covariance matrix. Weighted linear regression is applied to allow for weighting by the number of successfully genotyped or imputed RVs within the region of interest. The significance testing is based on a likelihood ratio test which compares the weighted likelihoods of the fitted model against a null model where **β** = 0. The likelihood ratio test statistic has an approximate χ2 distribution with *K* degrees of freedom.

The type I error rate and power of the method have been tested under various scenarios with simulated phenotype and genotype data, and the results from these analyses are described in detail elsewhere [19].

## Availability and requirements

**Project name:** MARV

**Project home page:** https://github.com/ImperialStatGen/MARV

**Operating system(s):** UNIX

**Programming language:** C++

**Other requirements:** Standard Linux/UNIX build tools to compile the program.

License: BSD 3-Clause License

Any restrictions to use by non-academics: None

## Additional files

Additional file 1: marv_chr1-5.result. Original results files outputted from MARV from the case study using NFBC1966 data. (RESULT 17825 kb)
Additional file 2: marv_chr6-10.result. Original results files outputted from MARV from the case study using NFBC1966 data. (RESULT 12614 kb)
Additional file 3: marv_chr11-15.result. Original results files outputted from MARV from the case study using NFBC1966 data. (RESULT 11495 kb)
Additional file 4: marv_chr16-22.result. Original results files outputted from MARV from the case study using NFBC1966 data. (RESULT 14097 kb)

## Abbreviations

BIC: Bayesian information criterion
FI: Fasting insulin
GWAS: Genome-wide association studies
MAF: Minor allele frequency
MARV: Multi-phenotype analysis of rare variants
MPA: Multi-phenotype analysis
NFBC1966: Northern Finland Birth Cohort
RV: Rare variant
TG: Triglycerides
WHR: Waist-to-hip ratio

## References

[1] Schatz MC (2015). Biological data sciences in genome research. Genome Res.

[2] McVean GA, Altshuler DM, Durbin RM, Abecasis GR, Bentley DR, Chakravarti A, Clark AG, Donnelly P, Eichler EE, Flicek P, Gabriel SB, Gibbs RA, Green ED, Hurles ME, Knoppers BM, Korbel JO, Lander ES, Lee C, Lehrach H, Mardis ER, Marth GT, McVean GA, Nickerson DA, Schmidt JP, Sherry ST, Wang J, Wilson RK, Gibbs RA, Dinh H, Kovar C (2012). An integrated map of genetic variation from 1,092 human genomes. Nature.

[3] Walter K, Min JL, Huang J, Crooks L, Memari Y, McCarthy S, Perry JRB, Xu C, Futema M, Lawson D, Iotchkova V, Schiffels S, Hendricks AE, Danecek P, Li R, Floyd J, Wain LV, Barroso I, Humphries SE, Hurles ME, Zeggini E, Barrett JC, Plagnol V, Brent Richards J, Greenwood CMT, Timpson NJ, Durbin R, Soranzo N, Bala S, Clapham P (2015). The UK10K project identifies rare variants in health and disease. Nature.

[4] The Haplotype Reference Consortium [http://www.haplotype-reference-consortium.org/home]. Accessed 8 Feb 2017.

[5] Huang J, Howie B, McCarthy S, Memari Y, Walter K, Min JL, Danecek P, Malerba G, Trabetti E, Zheng H-F, Gambaro G, Richards JB, Durbin R, Timpson NJ, Marchini J, Soranzo N, UK10K Consortium (2015). Improved imputation of low-frequency and rare variants using the UK10K haplotype reference panel. Nat Commun.

[6] Lee S, Abecasis GR, Boehnke M, Lin X (2014). Rare-variant association analysis: Study designs and statistical tests. Am J Hum Genet.

[7] Galesloot TE, Van Steen K, Kiemeney LA, Janss LL, Vermeulen SH (2014). A comparison of multivariate genome-wide association methods. PLoS One.

[8] Amos CI, Laing A (1993). A comparison of univariate and multivariate tests for genetic linkage. Genet Epidemiol.

[9] Allison DB, Thiel B, St Jean P, Elston RC, Infante MC, Schork NJ (1998). Multiple phenotype modeling in gene-mapping studies of quantitative traits: power advantages. Am J Hum Genet.

[10] Banerjee S, Yandell BS, Yi NJ (2008). Bayesian quantitative trait loci mapping for multiple traits. Genetics.

[11] Kim S, Xing EP (2009). Statistical estimation of correlated genome associations to a quantitative trait network. PLoS Genet.

[12] Jiang C, Zeng ZB (1995). Multiple trait analysis of genetic mapping for quantitative trait loci. Genetics.

[13] Shriner D (2012). Moving toward system genetics through multiple trait analysis in genome-wide association studies. Front Genet.

[14] Zhao J, Thalamuthu A (2011). Gene-based multiple trait analysis for exome sequencing data. BMC Proc.

[15] Marttinen P, Gillberg J, Havulinna A, Corander J, Kaski S (2013). Genome-wide association studies with high-dimensional phenotypes. Stat Appl Genet Mol Biol.

[16] Wang Y, Liu A, Mills JL, Boehnke M, Wilson AF, Bailey-Wilson JE, Xiong M, Wu CO, Fan R (2015). Pleiotropy analysis of quantitative traits at gene level by multivariate functional linear models. Genet Epidemiol.

[17] Purcell S, Neale B, Todd-Brown K, Thomas L, Ferreira MA, Bender D, Maller J, Sklar P, de Bakker PIW, Daly MJ, Sham PC (2007). PLINK: a tool set for whole-genome association and population-based linkage analyses. Am J Hum Genet.

[18] Marchini J, Howie B, Myers S, McVean G, Donnelly P (2007). A new multipoint method for genome-wide association studies by imputation of genotypes. Nat Genet.

[19] Kaakinen M, Mägi R, Fischer K, Heikkinen J, Järvelin M-R, Morris AP, Prokopenko I. A rare variant test for high-dimensional data. Eur J Hum Genet. 2017. Under revision. 10.1038/ejhg.2017.90 PMC5513099 28537275

[20] Mägi R, Asimit JL, Day-Williams AG, Zeggini E, Morris AP (2012). Genome-wide association analysis of imputed rare variants: application to seven common complex diseases. Genet Epidemiol.

[21] Rantakallio P (1969). Groups at risk in low birth weight infants and perinatal mortality. Acta Paediatr Scand.

[22] Scott R, Lagou V, Welch RP, Wheeler E, Montasser ME, Luan J, Mägi R, Strawbridge RJ, Rehnberg E, Gustafsson S, Kanoni S, Rasmussen-Torvik LJ, Yengo L, Lecoeur C, Shungin D, Sanna S, Sidore C, Johnson PCD, Jukema JW, Johnson T, Mahajan A, Verweij N, Thorleifsson G, Hottenga J-J, Shah S, Smith AV, Sennblad B, Gieger C, Salo P, Perola M (2012). Large-scale association analyses identify new loci influencing glycemic traits and provide insight into the underlying biological pathways. Nat Genet.

[23] Sabatti C, Service SK, Hartikainen A-L, Pouta A, Ripatti S, Brodsky J, Jones CG, Zaitlen NA, Varilo T, Kaakinen M, Sovio U, Ruokonen A, Laitinen J, Jakkula E, Coin L, Hoggart C, Collins A, Turunen H, Gabriel S, Elliot P, McCarthy MI, Daly MJ, Järvelin M-R, Freimer NB, Peltonen L (2009). Genome-wide association analysis of metabolic traits in a birth cohort from a founder population. Nat Genet.

[24] Rosenbloom KR, Armstrong J, Barber GP, Casper J, Clawson H, Diekhans M, Dreszer TR, Fujita PA, Guruvadoo L, Haeussler M, Harte RA, Heitner S, Hickey G, Hinrichs AS, Hubley R, Karolchik D, Learned K, Lee BT, Li CH, Miga KH, Nguyen N, Paten B, Raney BJ, Smit AF, Speir ML, Zweig AS, Haussler D, Kuhn RM, Kent WJ (2015). The UCSC genome browser database: 2015 update. Nucleic Acids Res.

[25] Ganna A, Salihovic S, Sundström J, Broeckling CD, Hedman ÅK, Magnusson PKE, Pedersen NL, Larsson A, Siegbahn A, Zilmer M, Prenni J, Ärnlöv J, Lind L, Fall T, Ingelsson E (2014). Large-scale metabolomic profiling identifies novel biomarkers for incident coronary heart disease. PLoS Genet.

[26] Feitosa MF, Wojczynski MK, Straka R, Kammerer CM, Lee JH, Kraja AT, Christensen K, Newman AB, Province MA, Borecki IB (2014). Genetic analysis of long-lived families reveals novel variants influencing high density-lipoprotein cholesterol. Front Genet.

[27] Major JM, Yu K, Wheeler W, Zhang H, Cornelis MC, Wright ME, Yeager M, Snyder K, Weinstein SJ, Mondul A, Eliassen H, Purdue M, Hazra A, McCarty CA, Hendrickson S, Virtamo J, Hunter D, Chanock S, Kraft P, Albanes D (2011). Genome-wide association study identifies common variants associated with circulating vitamin E levels. Hum Mol Genet.

[28] Major JM, Yu K, Weinstein SJ, Berndt SI, Hyland PL, Yeager M, Chanock S, Albanes D (2014). Genetic variants reflecting higher vitamin e status in men are associated with reduced risk of prostate cancer. J Nutr.

[29] Cha S, Yu H, Park AY, Song KH (2014). Effects of apolipoprotein A5 haplotypes on the ratio of triglyceride to high-density lipoprotein cholesterol and the risk for metabolic syndrome in Koreans. Lipids Health Dis.

[30] Gaunt TR, Zabaneh D, Shah S, Guyatt A, Ladroue C, Kumari M, Drenos F, Shah T, Talmud PJ, Casas JP, Lowe G, Rumley A, Lawlor DA, Kivimaki M, Whittaker J, Hingorani AD, Humphries SE, Day IN (2013). Gene-centric association signals for haemostasis and thrombosis traits identified with the HumanCVD BeadChip. Thromb Haemost.

[31] Grallert H, Dupuis J, Bis JC, Dehghan A, Barbalic M, Baumert J, Lu C, Smith NL, Uitterlinden AG, Roberts R, Khuseyinova N, Schnabel RB, Rice KM, Rivadeneira F, Hoogeveen RC, Fontes JD, Meisinger C, Keaney JF, Lemaitre R, Aulchenko YS, Vasan RS, Ellis S, Hazen SL, Van Duijn CM, Nelson JJ, März W, Schunkert H, McPherson RM, Stirnadel-Farrant H, Psaty BM (2012). Eight genetic loci associated with variation in lipoprotein-associated phospholipase A2 mass and activity and coronary heart disease: Meta-analysis of genome-wide association studies from five community-based studies. Eur Heart J.

[32] Waterworth DM, Ricketts SL, Song K, Chen L, Zhao JH, Ripatti S, Aulcheko Y, Zhang W, Yuan X, Lim N (2014). Genetic variants influencing circulating lipid levels and risk of coronary artery disease. Atheroscler Thromb Vasc Biol.

[33] Suchindran S, Rivedal D, Guyton JR, Milledge T, Gao X, Benjamin A, Rowell J, Ginsburg GS, McCarthy JJ (2010). Genome-wide association study of Lp-PLA(2) activity and mass in the Framingham Heart Study. PLoS Genet.

[34] Surakka I, Horikoshi M, Mägi R, Sarin A-P, Mahajan A, Lagou V, Marullo L, Ferreira T, Miraglio B, Timonen S, Kettunen J, Pirinen M, Karjalainen J, Thorleifsson G, Hägg S, Hottenga J-J, Isaacs A, Ladenvall C, Beekman M, Esko T, Ried JS, Nelson CP, Willenborg C, Gustafsson S, Westra H-J, Blades M, de Craen AJM, de Geus EJ, Deelen J, Grallert H (2015). The impact of low-frequency and rare variants on lipid levels. Nat Genet.

[35] Tokoro F, Matsuoka R, Abe S, Arai M, Noda T, Watanabe S, Horibe H, Fujimaki T, Oguri M, Kato K, Minatoguchi S, Yamada Y (2015). Association of a genetic variant of the ZPR1 zinc finger gene with type 2 diabetes mellitus. Biomed Rep.

[36] O’Reilly PF, Hoggart CJ, Pomyen Y, Calboli FCF, Elliott P, Jarvelin M-R, Coin LJM (2012). MultiPhen: Joint model of multiple phenotypes can increase discovery in GWAS. PLoS One.

[37] Mägi R, Suleimanov YV, Clarke GM, Kaakinen M, Fischer K, Prokopenko I, Morris AP. SCOPA and META-SCOPA: software for the analysis and aggregation of genome-wide association studies of multiple correlated phenotypes. BMC Bioinformatics. 2017. Accepted. 10.1186/s12859-016-1437-3 PMC5225593 28077070

